# Use of mobile technology-based participatory mapping approaches to geolocate health facility attendees for disease surveillance in low resource settings

**DOI:** 10.1186/s12942-018-0141-0

**Published:** 2018-06-18

**Authors:** Kimberly M. Fornace, Henry Surendra, Tommy Rowel Abidin, Ralph Reyes, Maria L. M. Macalinao, Gillian Stresman, Jennifer Luchavez, Riris A. Ahmad, Supargiyono Supargiyono, Fe Espino, Chris J. Drakeley, Jackie Cook

**Affiliations:** 1 Department of Immunology and Infection, London School of Hygiene and Tropical Medicine, London, WC1E 7HT UK; 2 Centre for Tropical Medicine, Faculty of Medicine, Universitas Gadjah Mada, Jln, Teknika Utara, Barek, Yogyakarta, 55281 Indonesia; 3 Faculty of Medicine and Health Sciences, Universiti Malaysia Sabah, Jalan UMS, Kota Kinabalu, Sabah Malaysia; 4 Department of Parasitology, Research Institute for Tropical Medicine, Research Drive, Alabang, Muntilupa, 1781 Metro Manila Philippines; 5 Department of Parasitology, Faculty of Medicine, Universitas Gadjah Mada, Sekip Utara, Yogyakarta, 55281 Indonesia; 6 MRC Tropical Epidemiology Group, Department of Infectious Disease Epidemiology, London School of Hygiene and Tropical Medicine, London, WC1E 7HT UK

**Keywords:** Electronic data collection, mHealth, Geographical information systems, Surveillance, Mobile technology, Participatory mapping

## Abstract

**Background:**

Identifying fine-scale spatial patterns of disease is essential for effective disease control and elimination programmes. In low resource areas without formal addresses, novel strategies are needed to locate residences of individuals attending health facilities in order to efficiently map disease patterns. We aimed to assess the use of Android tablet-based applications containing high resolution maps to geolocate individual residences, whilst comparing the functionality, usability and cost of three software packages designed to collect spatial information.

**Results:**

Using Open Data Kit GeoODK, we designed and piloted an electronic questionnaire for rolling cross sectional surveys of health facility attendees as part of a malaria elimination campaign in two predominantly rural sites in the Rizal, Palawan, the Philippines and Kulon Progo Regency, Yogyakarta, Indonesia. The majority of health workers were able to use the tablets effectively, including locating participant households on electronic maps. For all households sampled (n = 603), health facility workers were able to retrospectively find the participant household using the Global Positioning System (GPS) coordinates and data collected by tablet computers. Median distance between actual house locations and points collected on the tablet was 116 m (IQR 42–368) in Rizal and 493 m (IQR 258–886) in Kulon Progo Regency. Accuracy varied between health facilities and decreased in less populated areas with fewer prominent landmarks.

**Conclusions:**

Results demonstrate the utility of this approach to develop real-time high-resolution maps of disease in resource-poor environments. This method provides an attractive approach for quickly obtaining spatial information on individuals presenting at health facilities in resource poor areas where formal addresses are unavailable and internet connectivity is limited. Further research is needed on how to integrate these with other health data management systems and implement in a wider operational context.

**Electronic supplementary material:**

The online version of this article (10.1186/s12942-018-0141-0) contains supplementary material, which is available to authorized users.

## Background

Infectious disease risks can be highly heterogeneous at fine spatial scales due to environmental, social and biological factors [1]. As infectious disease control programmes move towards elimination, it is increasingly important to identify and target foci of transmission areas and understand the factors that may contribute to disease persistence in these locations [2–4]. Disease reports aggregated at coarser spatial scales, such as district or regional levels, may not capture these differences in micro-epidemiology [5, 6].

Numerous studies have utilised global positioning system (GPS) technology to develop fine-scale maps of disease infection and exposure (e.g. [7, 8]), identify hotspots of disease transmission (e.g. [9, 10]) and target control measures (e.g. [11, 12]). These studies typically use population-based cross-sectional surveys including GPS coordinates for patient households or frequently visited locations to map disease risks. Alternatively, when household surveys are not feasible, convenience sampling approaches targeting easy access groups can be used to estimate risks in a population. Examples of these approaches include school-based surveys (e.g. [13, 14]) and surveys of clinic attendees (e.g. [15]). These methods may not fully capture risks in the wider population but are substantially more cost effective to implement and may be more feasible in low resource settings.

A key limitation of convenience sampling approaches is that the interviewer does not visit the patient household and therefore cannot collect GPS coordinates at the site. If formal address information is available for a region, the patient address can be used to identify the GPS coordinates. However, this type of information is often not available for many countries or high-risk groups, such as migrant or mobile populations. In these situations, other methods can be used to estimate locations of patient households, such as identifying the nearest landmark, clinic or school catchment area or using participatory mapping techniques in which the patient identifies the location of their house on a paper map [16, 17]. These methods can be used to yield maps of relatively high spatial accuracy however, digitising maps and data management may be time consuming.

To address this issue, we assessed the use of tablet- based applications to geo-locate patient households remotely. Tablets are widely used to administer questionnaires and collect health information electronically as well as to scan barcodes and track samples [18–21]. Digital data collection can improve data quality and completeness as well as increase efficiency of data cleaning and analysis [22]. While these applications are frequently used to record GPS coordinates of the current location, the utility for participatory mapping for health surveys has not currently been assessed. We evaluated multiple software programs for use in rural resource poor settings with no internet connectivity as part of a malaria elimination research project. As such, a core requirement was the ability to load satellite images for use offline. We aimed to (1) identify appropriate tablet-based applications and assess the functionality, cost and technical expertise required to set up and use the programs; and (2) assess the accuracy of data collected using offline maps for the selected application.

## Methods

### Study areas

We evaluated different software programs for use in malaria surveillance of clinic attendees in two rural sites in Southeast Asia: Rizal Muncipality, Palawan, Philippines (1256 km^2^, estimated population 50,100, 15 health facilities) and Kulon Progo Regency, Yogyakarta, Indonesia (586 km^2^, estimated population 430,500, 8 health facilities). These sites are targets of on-going research projects to enhance surveillance for malaria elimination aiming to establish, integrate and evaluate combinations of laboratory, clinical and epidemiological data collected during health facility surveys to estimate the magnitude and heterogeneity of malaria transmission. Kulon Progo Regency is the site of one of the few remaining foci of malaria transmission in Java Island, Indonesia and was chosen as epidemiologically representative of a pre-elimination area where researchers and local control programmes are actively working towards elimination for Indonesia’s national strategic plan for malaria. Rizal, Palawan was selected as representative of an area in the Philippines transitioning from reduction of disease burden to malaria elimination. Samples were collected from patients and companions attending health facilities and microscopy, molecular and serological methods were used to identify infections and characterise transmission intensity. Both sites had multiple health facilities with poor or no internet connectivity. For each site, significant landmarks such as clinics, mosques, churches and schools were identified by local personnel and geo-located using a handheld GPS (Garmin, USA). Other spatial data, such as locations of roads and administrative boundaries, were assembled from available sources including government departments, freely accessible geospatial databases and open source GIS platforms such as OpenStreet Map (www.openstreetmap.org) and Global Administrative Areas (GADM; www.gadm.org).

### Data collection methods and survey

**Fig. 1:**
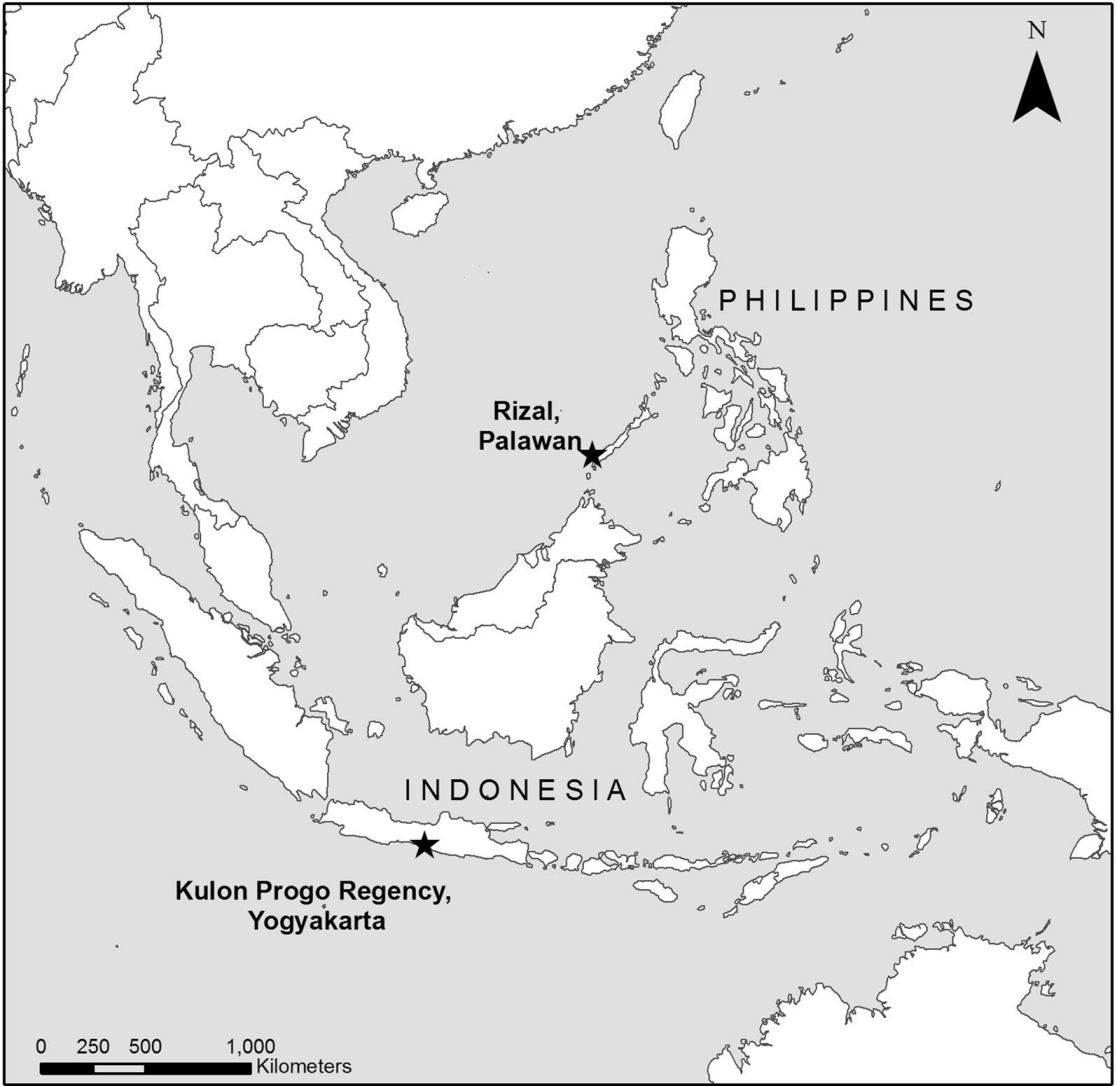
Study site locations

To develop data collection methods for these activities, we first evaluated multiple mobile-based data collection systems with the capacity to collect questionnaire data, GPS coordinates, and to take photographs of rapid diagnostic test results and scan barcodes used for sample tracking. For each software program, we set up a questionnaire as well as an offline map using best available satellite and GPS data for the health facility catchment area (Additional file 1). These questionnaires were tested by project staff in each site. All questionnaires were set up on Android tablets with 8 GB of internal memory and additional memory on external SD cards. Based on initial testing and map development, final data collection tools were designed using GeoODK and trialled in health facility surveys in the Philippines and Indonesia (Fig. 1). Maps were produced in Mapbox Studio, including high resolution satellite data, administrative boundaries and key landmarks and available census data.

**Fig. 2:**
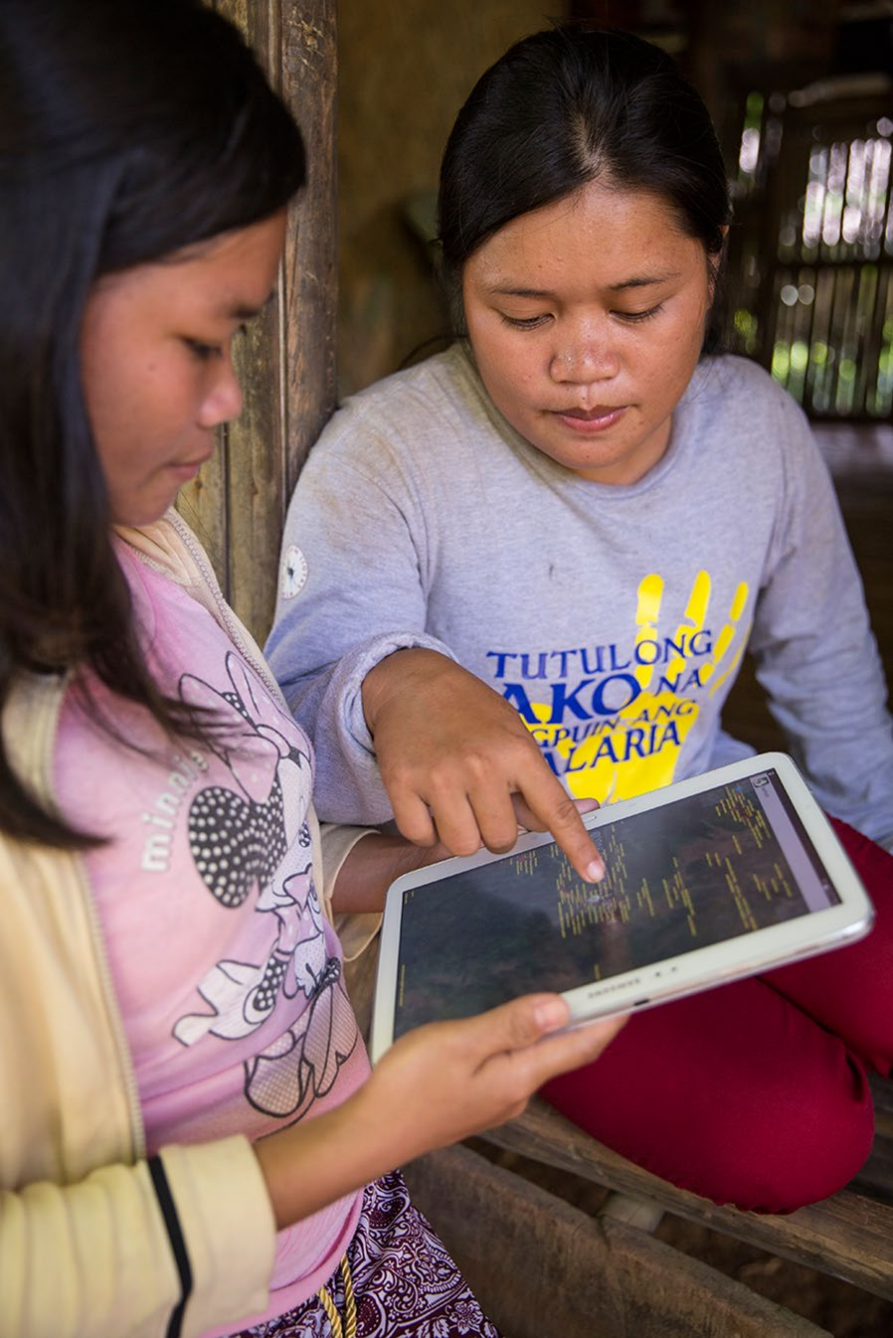
A health facility attendee identifies house location on a tablet in Rizal, Palawan, credit Joshua Paul

For multiple health facilities in each site, we conducted rolling cross sectional surveys of clinic attendees as part of larger malaria surveillance projects. During these surveys, consenting clinic attendees participated in a short questionnaire survey in Tagalog or Bahasa Indonesia and were asked to geo-locate their household using the digital maps provided. Initial 2 days training sessions were conducted for health facility personnel, followed by routine field supervision during the first week and regular meetings to identify any outstanding issues. Questionnaires were administered by the trained health facility personnel using Android-based tablets (Fig. 2). Data collected was checked for completeness and field and data management staff were interviewed on the ease of use and any issues with questionnaire or map data. Multiple health facilities from each site (3 facilities in Rizal and all 8 in Kulon Progo Regency) were selected to be representative of the data collected in each region, including the main regional health facility and several smaller satellite facilities in more remote areas. As this survey had an opportunistic sampling design, this population is not representative of the wider population in the study areas but rather individuals attending these health facilities. To assess the accuracy of reported GPS points, randomly selected households reporting to selected health facilities were followed up in both sites and GPS points of actual house locations were recorded using a handheld GPS. Although accuracy of the handheld GPS units could be impacted by poor satellite signal or high canopy or building coverage, the mean accuracy of these devices was within 5 m of the recorded household location and we considered this measurement the actual location of the household. Root mean square error of the Euclidean distance in meters between the actual and reported household locations was calculated to assess accuracy of participant’s estimates collected by tablets and identify factors affecting this accuracy.

## Results and discussion

### Software programs and characteristics

We initially identified three data collection programs capable of using offline maps: GeoODK (University of Maryland and International Institute for Applied Systems Analysis, College Park, USA), Survey123 for ArcGIS (Esri, Redlands, USA) and ePAL (Tripod Software, Salford, UK) (Table 1). GeoODK is an Android- based open source platform for form design, mobile data collection and data management system. Survey123 is a mobile data collection application which integrates into the Esri ArcGIS platform. ePAL is a custom-built application interfacing with other open source data collection systems (ODK and CommCare) to add capacity to use offline maps. While GeoODK was freely available, there were some developer costs for ePAL and ArcGIS Survey123 required the purchase of a software licence.

**Table 1:** Characteristics of software applications assessed

	GeoODK	ArcGIS Survey123	ePAL
Types of spatial data collected	Points, polygons and GPS tracks	Points, lines and polygons	Point
Able to load background maps offline	Yes	Yes	Yes
Format of offline maps	MBTile	ArcGIS Tile Package	PNG format tile dataset
Access to satellite imagery	External data only	Access to World Imagery and other ArcGIS layers	External data only
Storage space for imagery	Tablet internal memory	Tablet memory or external storage	Tablet memory or external storage
Questionnaire set up	XLS form	XLS form	Integrates with other data collection software
Integration	Integrates with barcode scanner and other software	Limited	Integrates with CommCare and ODK
Downloading data	Upload to server or download as XML file offline	Upload to server or download as SQLite database offline	Download as part of data from CommCare or ODK
Technical knowledge required	Some programming required to create MBTiles	Basic GIS knowledge only	Basic GIS knowledge only
Analysis	Summary statistics available from data on online server	Summary statistics available from data on online server	None
Cost and licencing	Free, open source software	Purchase of licence required (over USD 5000 for multiuser licence)	Limited purchase costs (less than USD 5000 developer costs)

Correspondingly, software programs had varying levels of technical support available. For GeoODK, part of the ODK open data kit, tutorials and manuals were available online in addition to active web forums for software developers and users. Product developers provided quick responses to technical queries and, in one instance, reviewed our questionnaires and maps to assist with troubleshooting. ArcGIS also had extensive user guides and tutorials available online. The software licence purchased included access to Esri technical support as well as online forums. While no formal documentation or support was available for ePAL, the software developers were available to address questions.

### Sources of satellite imagery and spatial data

In order for individuals to geo-locate their households, base maps must be assembled with sufficient spatial data on local geography and key landmarks. Freely available high resolution satellite data, such as Google Earth (www.google.com/earth), OpenStreetMap (www.openstreetmap.org) and Bing Maps (www.bing.com/maps), are increasingly used in public health to develop sampling frames [23, 24], collect spatially referenced disease data [25, 26] and target interventions [12, 27]. These data are usually of sufficient resolution to allow identification of individual houses and may contain further data on nearby points of interest. However, although these data can be freely accessed online, exporting imagery to raster datasets or other formats required for offline use is frequently covered by intellectual property agreements and may require user agreements or payments. Additionally, high resolution data is not always available in remote, sparsely populated areas and available data may not be temporally accurate, presenting a challenge in areas with high rates of change or following natural disasters.

Alternatively, very high resolution imagery is available through aerial photography or commercial satellite-based remote sensing sources, such as SPOT 6-7, Quickbird and IKONOS (www.digitalglobe.com). These data have resolutions of 1.5 m per pixel or less and have accurate data on the time of collection or can be tasked to collect data following significant changes. However, collecting these data can be prohibitively expensive in many low-income settings and processing and usage requires significant technical expertise. High resolution data may also be available through licensed software, for example Esri imagery through ArcGIS. Although accessing this imagery requires purchase of a software licence, high resolution imagery from aerial and satellite-based remote sensing is available for most of the world for offline use. These data are pre-processed and available in easy to use formats including metadata on the date of collection and temporal accuracy. Selecting the most appropriate imagery depends on the rates of land use change and development and availability of data for a particular region as well as the resources and technical expertise available.

In addition, the inclusion of geo-referenced information on key landmarks can help participants identify their houses or neighbourhoods [16]. This may include spatial point data on schools, clinics and other points of interest in addition to line or polygon data on roads, rivers and administrative districts. These datasets may be assembled from a range of sources such as government mapping departments, open source spatial data platforms (e.g. OpenStreet Map or GADM) or through collecting GPS data on the ground. In some instances, where previous community-based surveys have been conducted or censuses have collected GPS coordinates, point data may be available for individual houses. For each site, we used all available vector data, including any household head names, emphasising labels for commonly identified features.

### Setting up questionnaires and imagery

All software programs trialled used XLS forms to design questionnaires or, for ePal, integrated with other data collection software using XLS forms. However, each program required a different format for offline maps. ArcGIS Survey123 was the most user friendly option, allowing tile packages to be exported directly from ArcGIS with only basic GIS knowledge required. Both ePAL and GeoODK required additional processing time and expertise; ePAL required the creation of tiled PNG (Portable Network Graphics) datasets and GeoODK required MBTiles, a format storing tiled map data in SQLite databases which is commonly used by Android mapping applications. Production for both file types could be done using open source software such as Quantum GIS (www.qgis.org) and Mapbox Studio (www.mapbox.com) however MBTiles required some programming knowledge to correctly format maps.

For all formats, there were trade-offs between map resolution and speed. Producing high resolution maps resulted in large file sizes and consequently increased times to open maps on tablets. While both ArcGIS Survey123 and ePAL could store map files on either internal tablet memory or external SD cards, GeoODK could only use files stored on internal memory, limiting the possible size. For sites with more detailed spatial data relating to households and other landmarks, we reduced the resolution of the satellite data. If limited vector data were available, we increased the resolution but created multiple tiled datasets with smaller geographic areas to optimise rendering of maps on mobile devices.

### Field testing of data collection method

Based on the initial questionnaire testing, we chose to use GeoODK due to better integration of barcode scanners and other functionalities as well as faster loading of maps. Accuracy was not assessed for all software as all had similar map interfaces and accuracy was primarily dependent on the quality of the maps and the participant and interviewer abilities to use geographic information. GeoODK questionnaires and maps were set up on all tablets in the office while connected to the internet and data management staff were trained on setting up the questionnaire and downloading data offline. Training sessions were conducted to introduce fieldworkers to the use of the tablet and questionnaire; these field workers included community health workers and clinic staff, many of whom had not used electronic data collection methods or tablets prior to this work. Most fieldworkers were able to use the software effectively, although a few reported still preferring previously used paper data collection forms. Although there were some technical issues, such as forms freezing or crashing, the majority of data (over 99%) was complete and collected without any problems. Despite the inclusion of satellite imagery, most participants relied on names of household owners included on maps or labelled local landmarks rather than satellite imagery to locate the participant’s households. In some cases, when clinics were busy and maps were slow to load, fieldworkers did not wait for maps to load and fully zoom into an area, resulting in less accurate household geolocation; this issue was addressed by including maps with lower resolutions or smaller geographical areas which were faster to load.

An additional consideration is the availability of electricity; as not all clinics surveyed had reliable access to electricity or generators, we used external batteries or solar chargers in areas without constant power supplies. This did not result in the loss of any data but should be accounted for in budgeting and planning. Although data could be uploaded to an online server if an internet connection was available, the internet connection was poor and intermittent, resulting in the loss of data when the connection was interrupted during upload. Instead, all data was downloaded offline by copying XML files from the tablet memory to office computers. While GeoODK had functions to quickly produce summary statistics from data uploaded to the online server, we used R statistical programming language to read, merge and produce summary statistics for XML files (R statistical software, www.R-project.org).

### Accuracy of tablet-based geo-location strategies

**Fig. 3:**
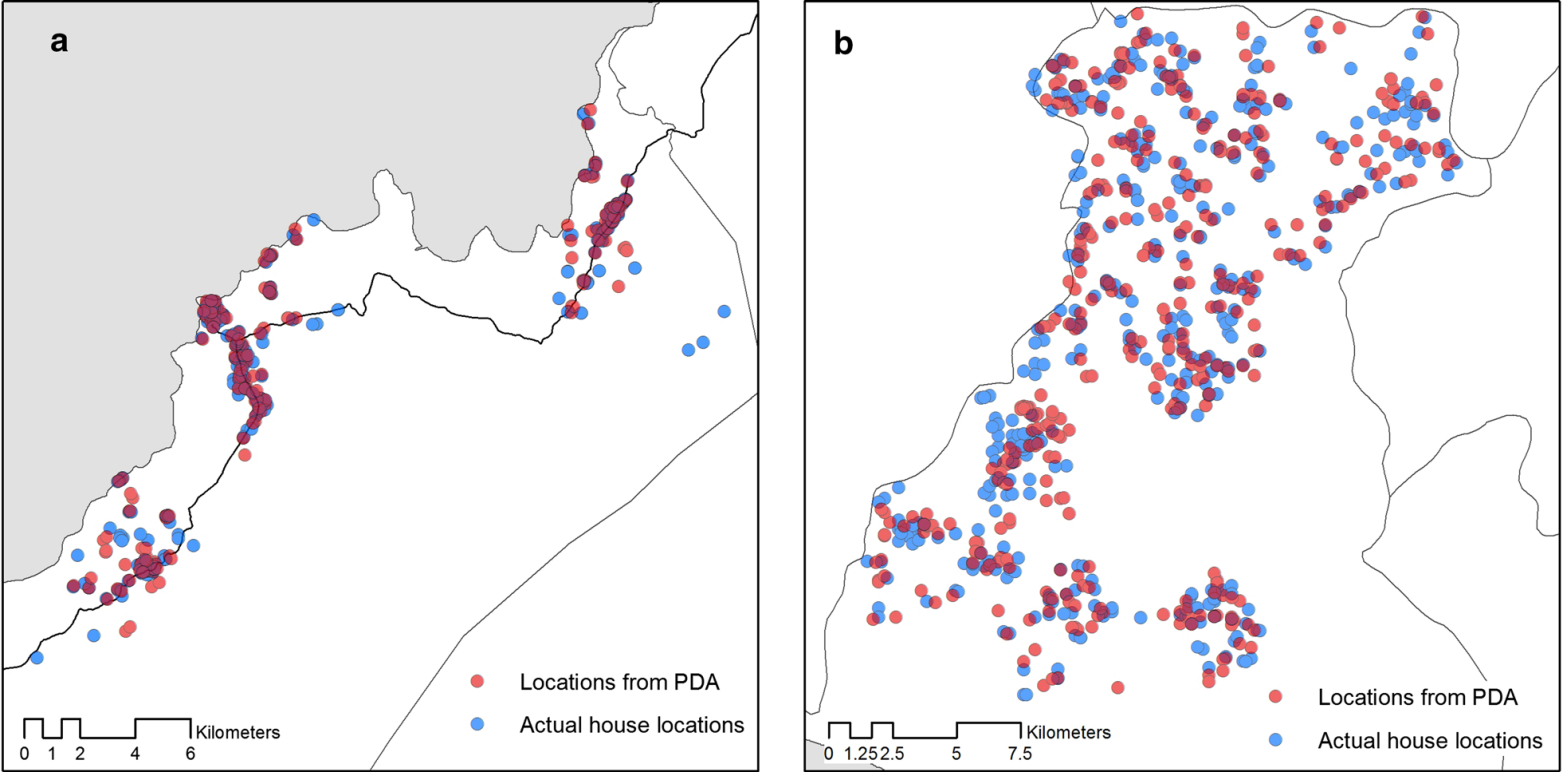
Comparison between house locations collected by tablet and actual house locations in **a** Rizal, Palawan. **b** Kulon Prugo Regency, Yogyakarta

To assess the accuracy of reported coordinates, we manually traced and recorded GPS points for 203 households in Rizal, Palawan and 400 households in Kulon Prugo Regency, Yogyakarta (Fig. 3). All households could be identified by fieldworkers using the name and locations collected by tablet and all households were located to their correct logistical unit used for interventions by the malaria control programme (sitios in the Philippines and desain Indonesia). Within these selected households, participants included 112 women and 91 men with a median age of 11 (range under 1–84 years) in Rizal, Palawan and 259 women and 143 men with a median age of 42 (range under 1–80 years) in Kulon Progo. In Rizal, 59 individuals had fever and 3 individuals were identified as malaria positive by microscopy while 34 individuals were febrile and 5 microscopy positive malaria cases were identified in Kulon Progo.

**Fig. 4:**
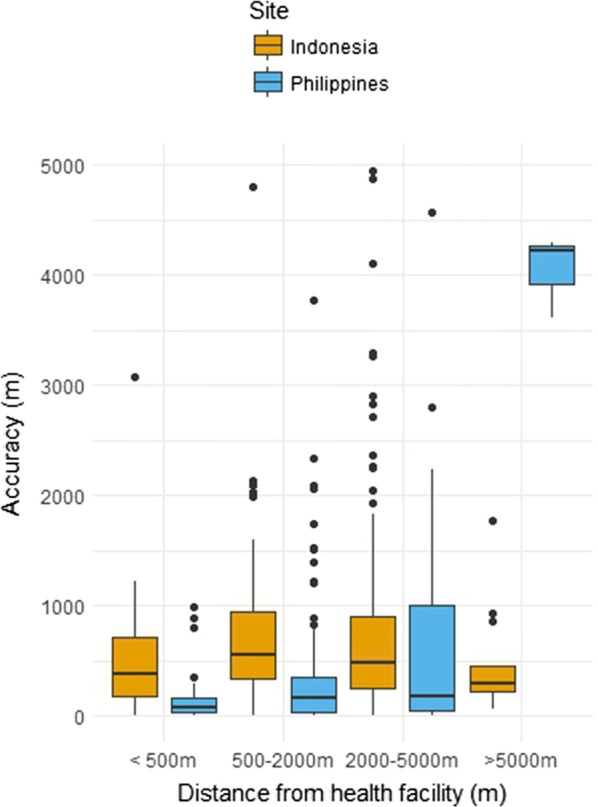
Spatial error of reported household locations by distance from health facility

The median distance between house locations and points recorded by the tablet was 116 m (IQR 42–368) in Rizal and 493 m (IQR 258–886) in Kulon Progo Regency. Root mean squared error was 895 and 702 m for Rizal and Kulon Progo Regency respectively. While most locations recorded by tablet were fairly accurate, a minority of points (6% in Rizal and 5% in Kulon Progo Regency) were over 2 km away from the actual house, primarily in areas where few landmarks were recorded. Although there was no clear relationship between accuracy of reported house locations and distance from the health facility, data collected on households over 2 km from the health facility were less accurate overall (Fig. 4). As geo-referenced point data was not available for all landmarks, we assessed whether areas with higher population density (places likely to have more distinct landmarks) were associated with accuracy of reported points. Gridded population density at 100 m resolution was obtained from WorldPop [28]; population density was not correlated with accuracy of reported points (*p* value = 0.11). These data may be improved by the inclusion of higher resolution maps or improved spatial information on remote areas. However, despite these limitations, data collected was of sufficient quality to identify houses of all sampled health facility attendees and enabled accurate fine-scale mapping of participants for these areas.

To assess the variability in accuracy between different health facilities in the same site, we compared spatial accuracy in records collected at all 8 facilities in Kulon Progo Regency. The accuracy of the mapping exercise varied within the 8 health facilities, with the closest accuracy measured in Samigaluh 1 (RMSE 353 m), and the least accuracy found in Girimulyo 2 (RMSE 817 m). Moreover, the exercise was able to locate 50.3% (95% CI 45.3–55.2%) and 78.3% (95% CI 74.2–82.3%) of households within an accuracy of ≤ 500 and ≤ 1000 m, respectively. The highest proportion of households that were located within < 1000 m were Samigaluh 1 (97.9%, 95% CI 93.8–100%), whilst the lowest proportion of households correctly located were Kokap 2 (53.1%, 95% CI 38.9–67.2). Of households that were not located within 1000 m (n = 86), 40.7% were in Kokap district, 31.4% in Girimulyo, 11.6% in Samigaluh and 8.1% in Kalibawang and 8.1% in Pengasih district.

**Fig. 5:**
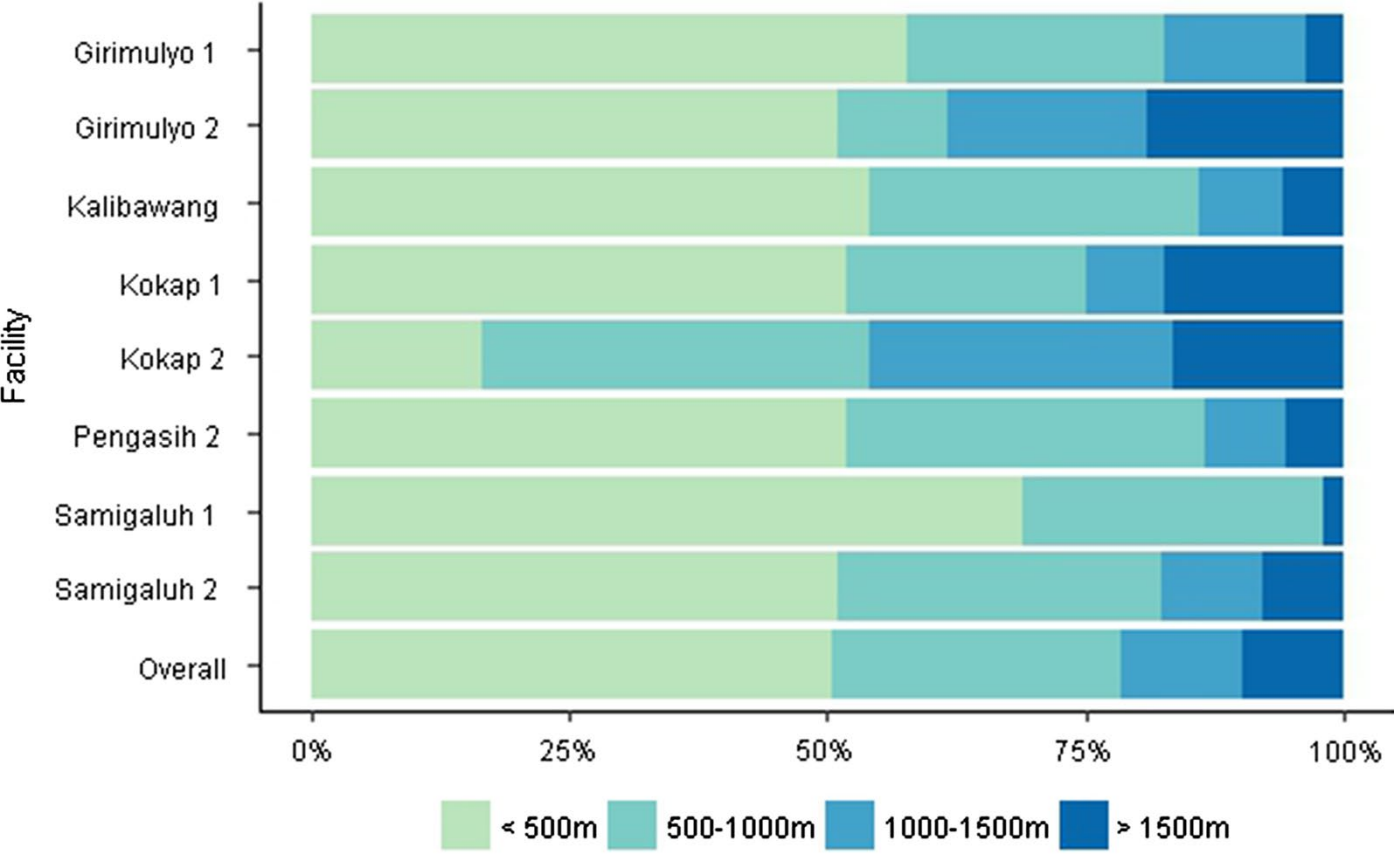
Accuracy by health facility in Kulon Progo Regency (proportion of households within specified distances)

While accuracy of GPS points was not significantly correlated with distance from the health facilities for the Regency overall (*p* value = 0.98), distance from the health facility was associated with decreased accuracy in the catchment area of Kokap 2 (*p* value = 0.003) (Fig. 5). This area is heavily forested and less densely populated, with very limited landmarks. Data suggests reported household locations in Kokap 2 were more accurate if they lived closer to the health facility or in close proximity to other landmarks such as mosques, schools or shops that were available on the map. In addition, the accuracy was higher in more populated health facility catchment areas where more landmarks were available.

## Conclusions

Tablet-based applications are an effective method of geo-locating participant households when it is not feasible to visit individual households. While numerous software platforms are available, selection should be based on the setting and resources available. Field testing of this software in the Philippines and Indonesia suggests data collection is sufficiently accurate to identify most households and would be appropriate for monitoring fine-scale spatial patterns of disease. Implementing this strategy could extend health facility capacity to remotely collect spatial information and monitor areas where infections are most regularly occurring. The rapid assessment of spatial representation of the population and any foci of infection or exposure can prevent spread of disease and support health programs to better target disease control and elimination activities.

The choice of software and spatial data to include should be guided by availability of data, technical expertise, required data resolution and resources. For sites with a stable internet connection and good coverage by Google Earth or other free imagery, software can be used with free online imagery. If no internet coverage is available, software such as GeoODK, ArcGIS Survey123 and ePAL can be used to incorporate offline maps. Licenced software such as ArcGIS Survey123 provides good access to high resolution imagery, technical support and requires only basic GIS knowledge to set up; however, this software requires the purchase of licences and may require additional costs. Alternatively, if other spatial data is available, open source software, such as GeoODK, or applications designed to interface with open source software, such as ePAL, can be used to include custom designed maps. This involves limited to no software costs but requires more technical expertise and may require additional costs for purchase of satellite imagery. The types of data to be collected, such as spatial points, line or polygon data, barcodes or images, should also be evaluated. For example, programmes with a sampling unit at an individual level is likely to require higher resolution point household locations while programmes targeting larger administrative units may require lower resolution polygon data. An additional consideration is whether geo-location data collection software will need to integrate with other data management systems, such as larger national health data management systems. As technology continues to develop, the functionalities of these programs as well as additional new software applications may continue to expand.

 The geolocation strategy tested in our study offers an alternative approach for obtaining spatial information from health-facility attendees in a setting that is typical for much of rural Southeast Asia and other parts of the world. The accuracy of the strategy in this setting improved in areas where more landmarks were available. This method could also be employed with other EAG (Easy Access Group) surveys such as school-based surveys that have been reportedly able to identify geographical variation in malaria transmission in different settings [13, 29–31]. Moreover, the GIS data collected in this study can be incorporated into a database that enables the display of information in the form of a basic map to enable reactive surveillance and other public health activities. In addition, this data should be linked to other environmental and spatial data so statistical analysis can identify associations between disease and environmental factors [4]. This can facilitate the identification of transmission hotspots are occurring and be used to target interventions [2].

Tablet-based geolocation strategies provide an important method of collecting spatial data in low resource settings when it is not feasible to visit patient households to directly collect GPS data and no formal address system is available. We have applied this approach in two settings in Southeast Asia, this approach is also being utilised in the Caribbean and African settings for both malaria and tuberculosis and therefore is applicable globally. While further research is needed to investigate the utility and feasibility of this method in a range of settings before implementing in a broader operational context, this study highlights the tools available and how these may be employed in low resource settings.

## Additional file

**Additional file 1.** Example questionnaire and associated data types.
