# Supplementary material for: Use of mobile technology-based participatory mapping approaches to geolocate health facility attendees for disease surveillance in low resource settings
Source: Int J Health Geogr. 2018 Jun 18;17:21. doi: 10.1186/s12942-018-0141-0 (PMC6006992; doi:10.1186/s12942-018-0141-0)

**Supplementary Information: Use of mobile technology-based participatory mapping approaches to geolocate health facility attendees for disease surveillance in low resource settings**

Kimberly M. Fornace*^&1^, Henry Surendra^&1,2^, Tommy Rowel Abidin^3^, Ralph Reyes^4^, Maria L. M. Macalinao^4^, Gillian Stresman^1^, Jennifer Luchavez^4^, Riris A. Ahmad^2^, Supargiyono Supargiyono^2,5^, Fe Espino^4^, Chris J. Drakeley^1^ & Jackie Cook^6^

1. Department of Immunology & Infection, London School of Hygiene and Tropical Medicine, WC1E 7HT, United Kingdom
2. Centre for Tropical Medicine, Faculty of Medicine, Universitas Gadjah Mada, Jln, Teknika Utara, Barek, Yogyakarta 55281, Indonesia
3. Faculty of Medicine and Health Sciences, Universiti Malaysia Sabah, Jalan UMS, Kota Kinabalu, Sabah, Malaysia
4. Department of Parasitology, Research Institute for Tropical Medicine, Research Drive, Alabang, Muntilupa, 1781 Metro Manila, Philippines
5. Department of Parasitology, Faculty of Medicine, Universitas Gadjah Mada, Sekip Utara, Yogyakarta 55281, Indonesia
6. MRC Tropical Epidemiology Group, Department of Infectious Disease Epidemiology, London School of Hygiene and Tropical Medicine, WC1E 7HT, United Kingdom

**Table SI1:** Example questionnaire and associated data types

| **Field ID** | **Question** | **Data type** |
| --- | --- | --- |
| date_consultation | 1) Date of Consultation: | Date/ time |
| barcode | 2) Participant's Barcode: | Barcode reader |
| I. TYPE AND LOCATION OF HEALTH FACILITY | | |
| type_facility | 1) Type of health facility: *(Select one)* | Field-list |
| oth_facility | 1-a) Other health facility: *(Select one)* | String |
| province | 2) Province: *(Select one)* | Field-list |
| municipality | 3) Municipality: *(Select one)* | Field-list |
| barangay | 4) Barangay: *(Select one)* | Field-list |
| sitio | 5) Sitio: *(Select one)* | Field-list |
| other_sitio | 5-a) Other Sitio: | String |
| II. PARTICIPANT'S PROFILE | | |
| participant_select | 1) Patient or Companion of Patient? (*Select one)* | Field-list |
| surname_name | 2) Last Name: | String |
| firstname_name | First Name: | String |
| middlename_name | Middle Initial: | String |
| suffix | Name Suffix, if any *(Jr. Sr., I, II, III, etc.)*: | String |
| gender | 3) Gender: *(Select one)* | Field-list |
| age | 4) Age |  |
| age_year | 4-a) Year: | Integer |
| age_months | 4-b) Months: | Integer |
| contact_number | 5) Contact Number: *(Optional)* | String |
| ethnicity | 6) Ethnicity: *(Select one)* | Field-list |
| oth_ethnicity | 6-a) Other name of Ethnic Group: | String |
| education | 7) Educational Attainment *(Select one):* | Field-list |
| occupation | 8) Current occupation (by industry): - Select all that apply | Field-list |
| oth_occupation | 8-a) Other occupation | String |
| III. RESIDENCE AND GPS COORDINATES | | |
| stay_length | 1) How long have you been living at your present address? *(Select one)* | Field-list |
| barangay_2 | 1-a) Barangay: (Answer without abbreviating) | Field-list |
| sitio_2 | 1-b) Sitio: *(Answer without abbreviating)* | String |
| gps_tag | 1-c) Has the Location of Primary Residence been identified? | Yes/ No |
| residence_primary | 1-d) Location of Primary Residence: | GPS coordinates |
| other_residence | 2) Do you have any other residence? | Yes/ No |
| visited_secondary | 2-a) If YES, Have you visited your other residence these past 4 weeks? | Yes/ No |
| barangay_3 | 2-b) Barangay: (Answer without abbreviating) | Field-list |
| sitio_3 | 2-c) Sitio: (*Answer without abbreviating)* | String |
| residence_secondary | 2-d) Location of Secondary Residence: | GPS coordinates |
| IV. TRAVEL, AND ACTIVITIES | | |
| travel | 1) Have you traveled anywhere in the past 4 weeks? | Yes/ No |
| places_visited1 | 1-a) If YES, what places have you visited? | Field-list |
| places_visited2 | Other places: | String |
| places_visited3 | Other places: | String |
| activities1 | 2) What activities did you do these past 2 weeks? | String |
| activities2 | Other activities: | String |
| activities3 | Other activities: | String |
| V. BEDNET OWNERSHIP | | |
| bednet_use | 1) Do you own a bed net? | Yes/ No |
| bednet_ins | 1-a) If YES, was the bednet treated with insecticide? | Yes/ No |
| bednet_use | 1-b) If YES, do you use the bednet? | Yes/ No |
| VI. HEALTH INFORMATION | | |
| temp_fever | 1) What is your temperature on the day of consultation? *(Select one)* | Field-list |
| axillary_temperature | 1-a) After getting you axillary temperature, does it indicate of having a fever? | Yes/ No |
| fever_length | 1-b) If **YES,** how many day/s has it been since the onset of your fever? (*Select one)* | Field-list |
| fever_length_meds | 1-c) Did you take any medicine for your fever? | Field-list |
| fever_drugs | 1-d) If YES, what fever medicine did you take? | Field-list |
| oth_fdrugs | Other fever medicine: | Field-list |
| oth_symptoms_select | 2) Are you experiencing any other symptom? | Yes/ No |
| symptoms_select | 2-a) If YES, what other symptoms are you experiencing? (*Select all that apply)* | Field-list |
| symptoms_select_other1 | Other symptoms: | String |
| symptoms_select_other2 | Other symptoms: | String |
| symptoms_select_other3 | Other symptoms: | String |
| symptoms_length | 2-b) How long have you had these symptoms? (*Select one)* | Field-list |
| VII. DIAGNOSTIC TESTS | | |
| filter_paper | 1) Sample specimen for Filter Paper collected? | Yes/ No |
| bsmp | 2) Sample specimen for Blood Film collected? | Yes/ No |
| rdt | 3) Was Malaria Rapid Diagnostic Test (RDT) performed? | Yes/ No |
| VIII. RAPID DIAGNOSTIC TEST RESULT | | |
| blood_examined | 1) Date of Blood Examined: | Date/ time |
| rtd_type | 2) Type of RDT Kit used: | Field-list |
| rdt_result | 3) RDT Result: | Field-list |
| rdt_positive | 3-a) Type of Malaria Infection: | Field-list |
| image_RDT | 3-b) Take a picture of the RDT kit result. | Image |
| rdt_treatment | 4) Treatment for Malaria-infected individual: | field-list |
| referred | 5) Reason for referral: | field-list |

**Figure SI1:** Screenshots of GeoODK questionnaire on Android tablet

1. Offline map loaded on GeoODK questionnaire


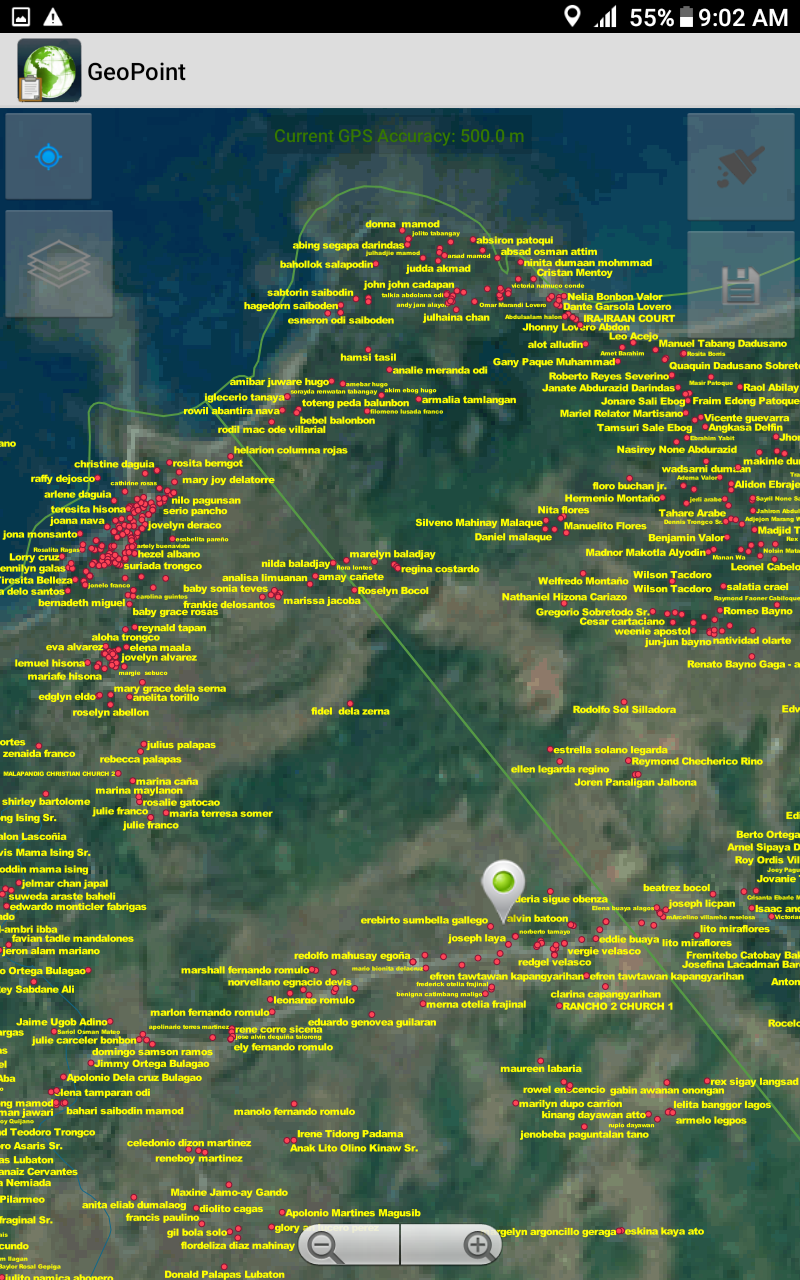


1. GPS coordinates collected using offline map


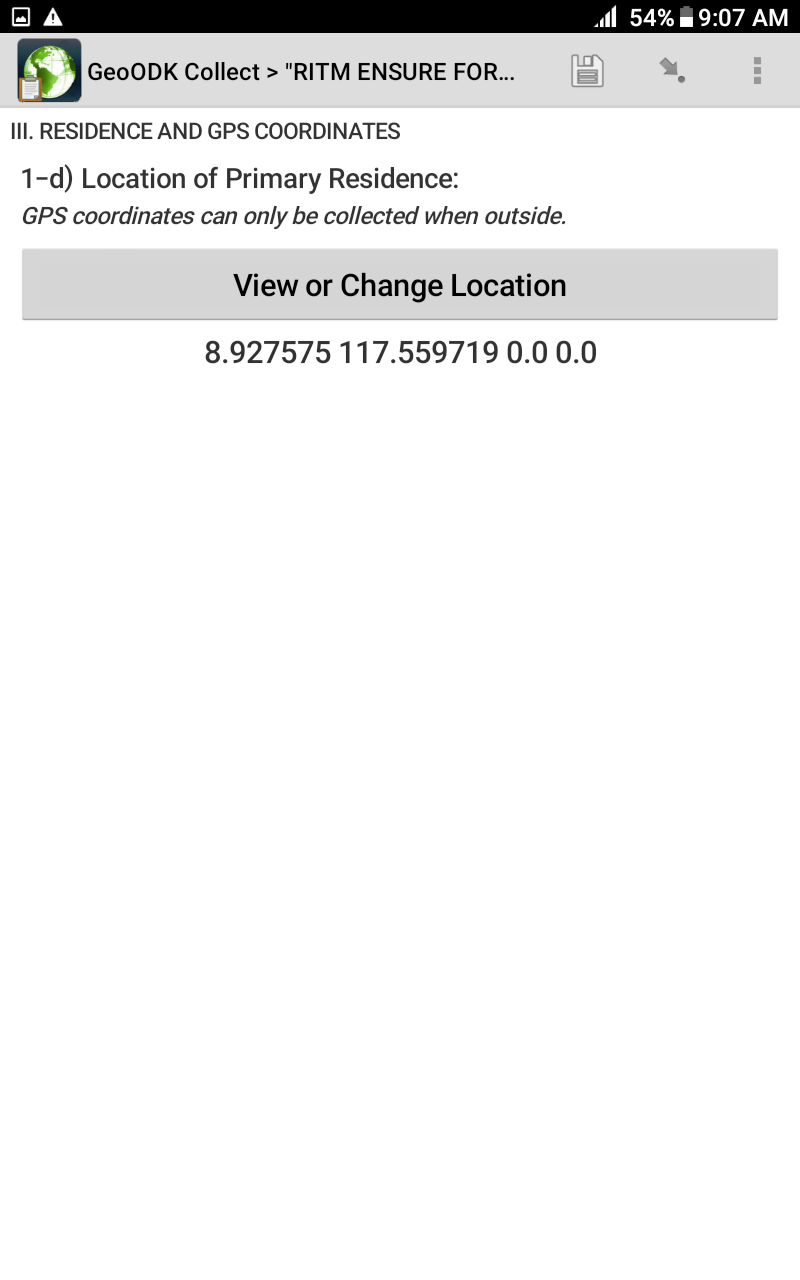


1. Barcode scanner used to scan sample barcode labels


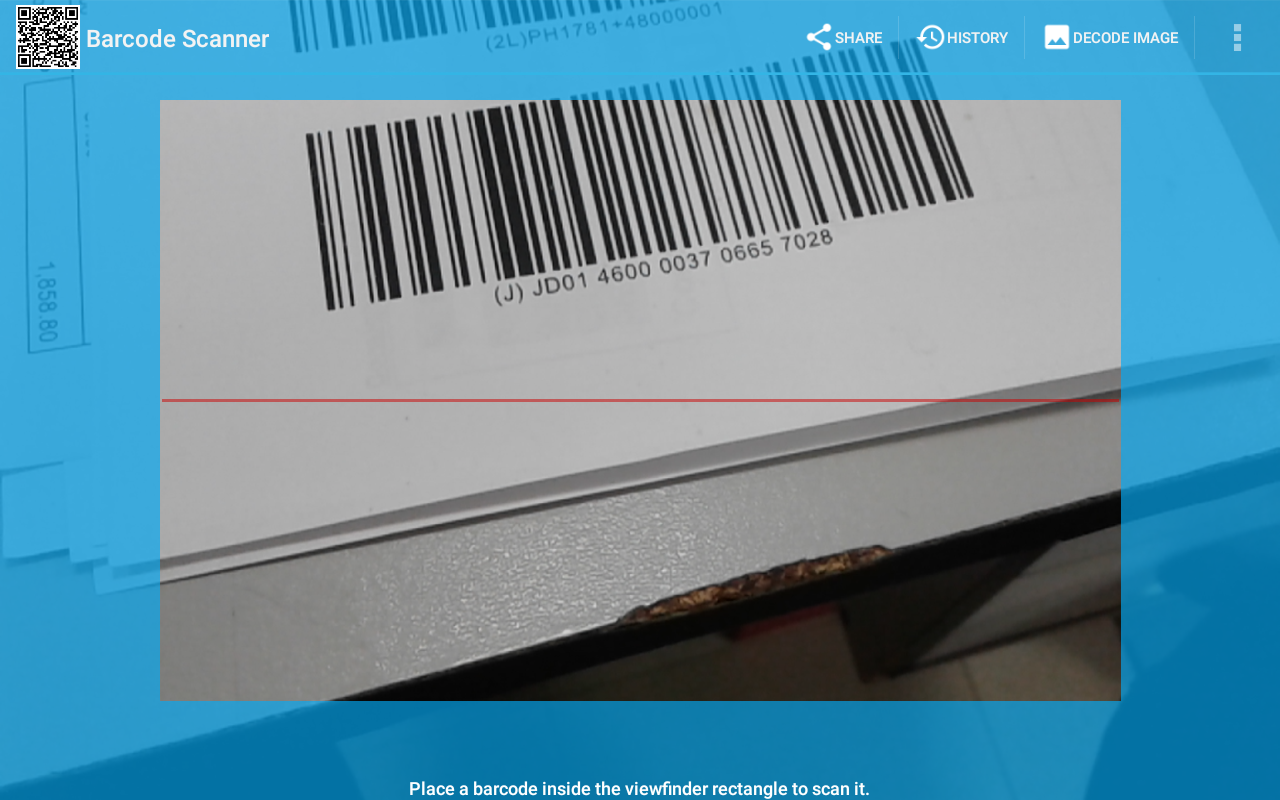


1. Example of collection of multiple choice questionnaire data


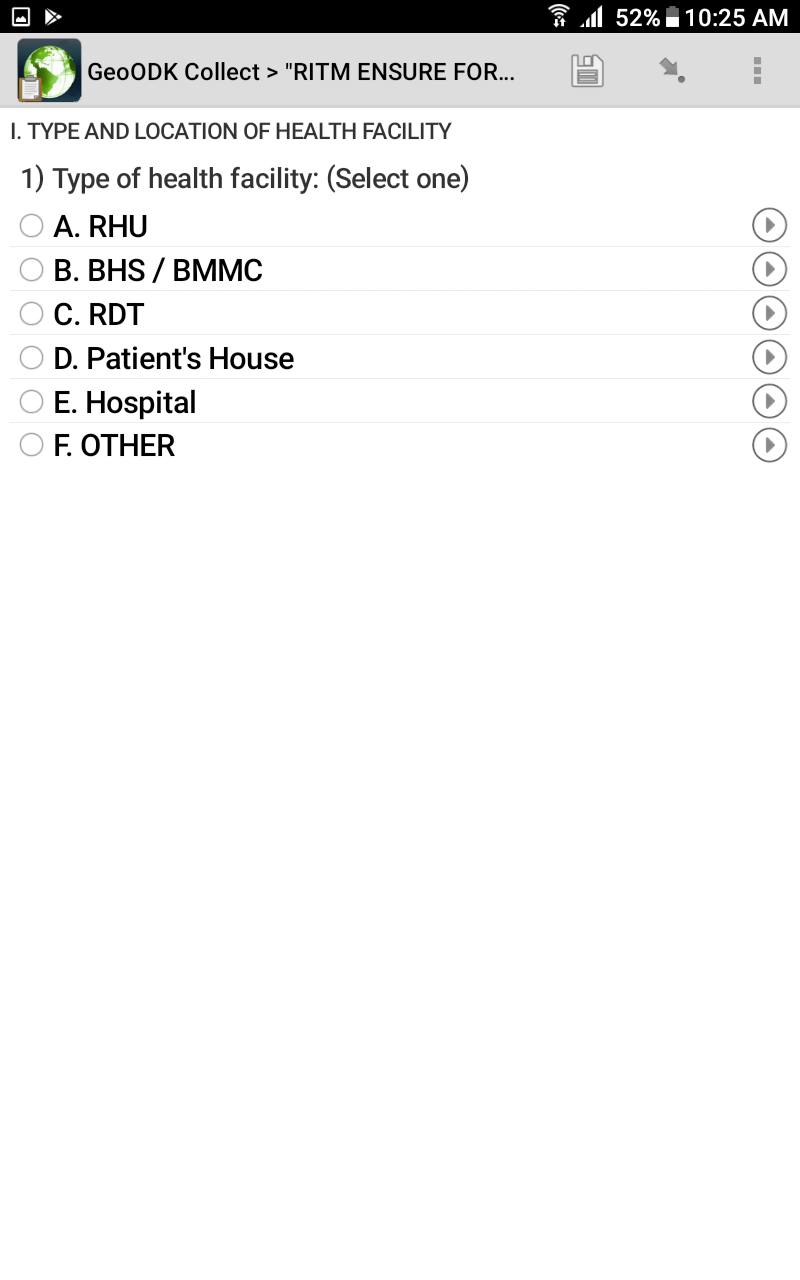

Supplement: Supplementary file 1 — Additional file 1. Example questionnaire and associated data types. [file 12942_2018_141_MOESM1_ESM.docx]
